# Variations in capillary and serum lactates levels based on different etiologies of septic patients in the emergency department

**DOI:** 10.3389/fmed.2025.1536148

**Published:** 2025-04-16

**Authors:** Matteo Guarino, Benedetta Perna, Giacomo Maroncelli, Paolo Baldin, Chiara Donati, Francesco Luppi, Alice Eleonora Cesaro, Chiara Pesci, Anna Costanzini, Martina Maritati, Paolo Clavenzani, Carlo Contini, Roberto De Giorgio, Michele Domenico Spampinato

**Affiliations:** ^1^Department of Translational Medicine, St. Anna University Hospital of Ferrara, University of Ferrara, Ferrara, Italy; ^2^Department of Emergency, St. Anna University Hospital of Ferrara, Ferrara, Italy; ^3^Infectious Diseases Unit, Department of Medical Sciences, University of Ferrara, Ferrara, Italy; ^4^Department of Veterinary Medical Sciences, University of Bologna, Bologna, Italy

**Keywords:** diastolic shock index, etiology, lactates, neutrophil-to-lymphocyte ratio, sepsis, septic shock

## Abstract

**Introduction:**

Sepsis is a life-threating and time-depending condition. This study examined the association between sepsis etiology and variations in capillary and serum lactate levels, neutrophil-to-lymphocyte ratio, and diastolic shock index in emergency department patients.

**Methods:**

This study, conducted between 2021 and 2022 at the Emergency Department of Ferrara, included the following criteria: (i) clinical suspect of infectious disease; (ii) qSOFA ≥2; (iii) age ≥18 years; (iv) signed informed consent. Etiologies were: (i) negative cultures (NC); (ii) Gram positive (GP); (iii) Gram negative (GN); (iv) fungal infections (FI).

**Results:**

Among the 200 included patients, 104 (52.0%) had NC, 36 (18.0%) GP, 53 (26.5%) GN and 7 (3.5%) FI. CLs (*p* = 0.006) and SLs (*p* < 0.001) were different according to etiology being higher in GP infections. NLR (*p* = 0.035) was higher in GN infections, while DSI (*p* = 0.008) increased in FI. Mortality was not influenced by the etiology.

**Conclusion:**

All parameters differed according to sepsis etiology, thus improving early prediction of sepsis etiology and its pharmacological management.

## Introduction

Sepsis is a global health priority with a significant increase of incidence worldwide (1, 2). From a pathogenetic standpoint, this life-threatening and time-dependent organ dysfunction results from an altered host response to a wide range of infections (1). The lack of reliable biomarkers, the limited usefulness of diagnostic scores (e.g., quick Sequential Organ Failure Assessment [qSOFA], and National Early Warning Score-2 [NEWS-2]), and its multifaceted treatment (i.e., antimicrobials, fluid replacement, vasopressors and oxygen) make sepsis a clinical challenge (3, 4). Among various etiologies, pulmonary infections are considered the most prevalent ones followed by urinary tract, abdominal, endocarditis, soft tissue, bone and central nervous system infections (5). However, up to 50% of septic cases shows negative blood cultures despite clinical suspicion of infection (5, 6). This latter aspect requires a careful clinical evaluation to explore alternative sources of infection and guide appropriate antimicrobial therapy (6). Following pathogen infection, the host immune system initiates a cascade of inflammatory mediators, leading to vasodilation, increased vascular permeability, and altered microcirculatory blood flow. These changes contribute to tissue hypoperfusion and cellular hypoxia, prompting anaerobic metabolism and subsequent lactate production. Furthermore, mitochondrial dysfunction exacerbates lactate accumulation by impairing aerobic respiration and oxidative phosphorylation. Therefore, lactate levels can be used to assess sepsis severity and prognosis, reflecting the underlying physiopathological processes of tissue hypoperfusion, cellular dysfunction, and mitochondrial injury (7). In a previous study, we demonstrated that capillary lactates (CLs) can be a reliable tool for the early identification of septic patients at high risk of 48-h and 7-day mortality (7). Furthermore, in the latest years, both neutrophil-to-lymphocyte ratio (NLR) (8) and diastolic shock index (DSI) (9) have been also reported as good predictors of fatal outcome in septic patients.

The main goal of this study was to assess whether variations in CLs and serum lactates (SLs) levels vary according to sepsis etiology in patients admitted to the Emergency Department (ED). The secondary aim is to evaluate whether NLR and DSI could statistically differ depending on etiology.

## Materials and methods

This is a monocentric, observational and prospective study performed between October 2021 and May 2022 at the Emergency Department (ED) of the St. Anna University Hospital of Ferrara, Italy. Inclusion criteria were: (i) suspect of infectious disease; (ii) age ≥18 years; (iii) qSOFA ≥ 2; (iv) a signed informed consent obtained from each involved patient (or their relatives in case of severe clinical conditions). Patients found to be non-septic were excluded. This protocol was proposed before the publication of 2021 sepsis guidelines (3), therefore qSOFA has been used as severity screening tool. Before starting the enrollment, specific tests were performed to determine the sample size needed to achieve statistical significance in the proposed outcomes.

The following data were retrieved for each patient: (i) capillary and arterial blood samples to assess lactate levels; (ii) NEWS-2; (iii) laboratory tests (i.e., blood cells count, creatinine, bilirubin, PaO_2_/FiO_2_ ratio); (iv) sepsis focus (i.e., respiratory, urinary, abdominal, miscellaneous and undefined); (v) microbiological etiologies defined as negative cultures (NC), Gram positive (GP), Gram negative (GN) and fungal infection (FI); (vi) final diagnosis. Patients’ comorbidities were assessed using the Charlson Comorbidity Index (CCI). To account for the potential influence of age as a confounding factor, we performed an age-adjusted analysis by dividing patients into three age groups: <65 years, 65–80 years, and >80 years.

As explained in our previous study (10), CLs were assessed on admission at ED with a LactateProTM2^®^, Arkray Global Business Inc., Kyoto, Japan. The study was approved by the local Ethics Committee (Protocol No. 447/2021/Disp/AOUFe).

### Statistical analysis

Continuous data were described using the median and interquartile range (IQR), while categorical data were presented as counts and percentages. For comparisons among at least three independent groups, the Kruskal–Wallis test was used for continuous data, while Fisher’s exact test was used for two-level categorical variables and the chi-square test for independence was used for categorical variables with more than two levels. The significance level was set for *p* < 0.05. False discovery rate correction was applied for multiple comparisons. Data analysis was conducted using the jamovi project (2024), jamovi (version 2.5) (available at https://www.jamovi.org).

## Results

A total of 212 patients were initially considered, of whom 12 were excluded as they did not meet the diagnostic criteria for sepsis. The final analysis included 200 patients (112 females and 88 males) with a median age of 85 years (IQR 74–90) and a median Charlson Comorbidity Index (CCI) of 4 (IQR 1–4). No sex differences were reported (*p* = 0.33). Patients were classified based on sepsis microbiological etiology into four groups: negative cultures (NC, 52.0%); Gram-positive infections (GP, 18.0%); Gram-negative infections (GN, 26.5%); and fungal infections (FI, 3.5%). Patients with fungal infections were significantly older (median age: 94 years, IQR 90–95) compared to the other groups (*p* < 0.001). Mean arterial pressure (MAP) was significantly lower in the GP, GN, and FI groups compared to NC (*p* = 0.003), and diastolic shock index (DSI) was highest in the FI group (*p* = 0.008). Capillary and serum lactate levels varied significantly between groups (*p* = 0.006 and *p* < 0.001, respectively), with the highest values observed in GP and GN infections. Additionally, neutrophil-to-lymphocyte ratio (NLR) was significantly different among groups (*p* = 0.035), with the highest median NLR in the GN group (12, IQR 7–25). The incidence of respiratory and urinary sepsis foci differed significantly across groups (*p* < 0.001). Both 48-h (*p* = 0.72) and in-hospital mortality (IHM) (*p* = 0.70) were not influenced by microbiological etiologies even if they were higher in the GP group. Other results were summarized in Table 1.

**Table 1:** Main results highlighting the relationship between patients’ clinical/laboratory features and sepsis microbiological etiologies.

Features	NC 104 (52.0%)	GP 36 (18.0%)	GN 53 (26.5%)	FI 7 (3.5%)	*p*
Male sex, %	45 (43.3%)	12 (33.3%)	28 (52.8%)	3 (42.8%)	0.33
Age, years	87 (81, 91)	84 (78, 87)	83 (76, 87)	94 (90, 95)	<0.001
CLs, mmol/L	4.5 (3.0, 7.9)	9.0 (5.3, 12.7)	6.0 (4.0, 11.4)	4.3 (3.4, 6.6)	0.006
SLs, mmol/L	1.0 (0.6, 1.4)	1.8 (0.8, 2.6)	1.7 (1.1, 3.3)	1.6 (1.2, 3.1)	<0.001
MAP, mmHg	80 (73, 91)	73 (70, 83)	73 (67, 80)	73 (58, 73)	0.003
HR, bpm	90 (80, 100)	105 (80, 115)	97 (82, 106)	88 (78, 106)	0.21
RR, apm	24.0 (22.0, 28.0)	27.0 (22.0, 30.0)	24.0 (22.0, 28.0)	23.0 (22.0, 28.5)	0.20
Body temperature, °C	37.0 (36.5, 38.0)	37.9 (36.6, 38.2)	37.5 (36.5, 38.4)	37.5 (37.0, 37.9)	0.55
GCS	13.0 (10.0, 14.0)	12.5 (9.0, 15.0)	13.0 (11.0, 14.0)	12.0 (9.5, 13.5)	0.79
SpO_2_, %	96 (94, 98)	97 (95, 98)	96 (94, 98)	95 (91, 97)	0.15
FiO_2_, %	21 (21, 28)	26 (21, 40)	21 (21, 21)	21 (21, 21)	0.022
DSI	1.36 (1.15, 1.65)	1.55 (1.28, 1.96)	1.63 (1.29, 2.00)	1.90 (1.38, 2.59)	0.008
NEWS-2	8.0 (4.8, 10.0)	9.0 (7.8, 12.0)	8.0 (5.0, 10.0)	9.0 (7.0, 9.5)	0.077
SOFA	4.0 (3.0, 6.0)	4.5 (3.0, 7.0)	5.0 (4.0, 6.0)	5.0 (4.0, 5.5)	0.089
pH	7.43 (7.39, 7.48)	7.42 (7.38, 7.47)	7.46 (7.40, 7.48)	7.46 (7.44, 7.50)	0.14
P/F	325 (264, 394)	351 (244, 398)	342 (309, 393)	326 (298, 375)	0.60
NLR	8 (5, 12)	11 (6, 20)	12 (7, 25)	10 (4, 22)	0.035
Platelets, U/mmc	230 (161, 284)	235 (162, 295)	196 (147, 275)	252 (220, 272)	0.49
Creatinine, mg/dL	1.09 (0.77, 1.69)	1.26 (0.87, 1.77)	1.84 (1.20, 2.63)	1.49 (1.32, 1.64)	0.004
Total bilirubin, mg/dL	0.75 (0.52, 1.11)	0.80 (0.53, 1.21)	0.80 (0.52, 1.11)	0.96 (0.40, 1.13)	>0.99
CCI	2 (1, 4)	2 (1, 4)	3 (2, 5)	2 (2, 4)	0.17
Sepsis focus					<0.001
Respiratory, %	37 (35.6%)	12 (33.3%)	8 (15.1%)	1 (14.3%)	
Urinary, %	34 (32.7%)	8 (22.2%)	37 (69.8%)	4 (57.1%)	
Abdominal, %	10 (9.6%)	2 (5.6%)	2 (3.8%)	1 (14.3%)	
Miscellaneous, %	11 (10.6%)	9 (25.0%)	4 (7.5%)	1 (14.3%)	
Undefined, %	12 (11.5%)	5 (13.9%)	2 (3.8%)	0 (0%)	
48-h Mortality, %	9 (8.7%)	5 (14%)	5 (9.4%)	0 (0%)	0.72
IHM, %	30 (29%)	12 (33%)	12 (23%)	2 (29%)	0.70

Continuous data were described using the median and interquartile range (IQR), while categorical data were presented as counts and percentages (%). CCI, Charlson Comorbidity Index; CLs, capillary lactates; DBP, diastolic blood pressure; DSI, diastolic shock index; ED, emergency department; FI, fungal infection; FiO_2_, fraction of inspired oxygen; GCS, Glasgow Coma Scale; GN, Gram negative; GP, Gram positive; HR, heart rate; IHM, in-hospital mortality; IQR, interquartile range; MAP, mean arterial pressure; NC, negative culture; NEWS-2, National Early Warning Score-2; NLR, neutrophil-to-lymphocyte ratio; P/F, PaO_2_/FiO_2_ ratio; RR, respiratory rate; SBP, systolic blood pressure; SLs, serum lactates; SOFA, Sequential Organ Failure Assessment; SpO_2_, saturation of peripheral oxygen.

To further explore the potential influence of age as a confounding factor, we performed an age-adjusted analysis, dividing patients into three age groups: <65 years, 65–80 years, and >80 years. The results, summarized in Table 2, indicated that there were observable trends in the values of CLs, serum SLs, DSI, and CCI across different age groups, although the small sample size limited the statistical significance in some cases. In patients younger than 65 years, the CCI was significantly higher in those with GN infections. For the 65–80 years age group, SL levels were significantly higher in patients with GN infections compared to other groups, and although CL levels were higher in both GP and GN infections, the difference was not statistically significant. In patients older than 80 years, CL levels were significantly higher in those with GP infections, while the CCI was significantly lower in patients with NC. No significant differences in 48-h or in-hospital mortality were observed across the different age groups, a finding consistent with our overall results.

**Table 2:** Age-adjusted analysis of CLs, SLs, DSI, NLR, CCI, and mortality outcomes across three age groups: <65 years, 65–80 years, and >80 years.

	Age <65 years				Age 65, 80 years					Age >80 years				
	NC *n* = 2 (28.6%)	GP *n* = 3 (42.9%)	GN *n* = 2 (28.6%)	*p*	NC *n* = 19 (42.2%)	GP *n* = 7 (15.6%)	GN *n* = 18 (40%)	FI *n* = 1 (2.2%)	*p*	NC *n* = 83 (56.1%)	GP *n* = 26 (17.6%)	GN *n* = 33 (22.3%)	FI *n* = 6 (4.1%)	*p*
CLs	5 (4, 5)	16 (9, 25)	13 (11, 15)	0.327	4 (3, 6)	10 (5, 18)	10 (4, 17)	5 (5, 5)	0.162	5 (3, 8)	9 (6, 11)	5 (3, 9)	4 (3, 9)	0.034
SLs	1 (0, 1)	4 (1, 21)	2 (2, 3)	0.327	1 (1, 1)	2 (1, 6)	3 (2, 6)	1 (1, 1)	0.005	1 (1, 2)	2 (1, 2)	1 (1, 2)	2 (1, 4)	0.084
DSI	1.46 (1.29, 1.63)	2.5 (2.34, 3.25)	2.13 (2, 2.25)	0.840	1.33 (1.14, 1.75)	1.7 (1.33, 1.91)	1.51 (1.2, 2.12)	1.93 (1.93, 1.93)	0.233	1.36 (1.14, 1.6)	1.5 (1.2, 1.83)	1.62 (1.38, 1.75)	1.68 (1.28, 3.25)	0.663
NLR	5 (2, 9)	7 (3, 9)	7 (3, 9)	0.531	8 (5, 12)	9 (4, 14)	10 (7, 23)	10 (6, 22)	0.111	8 (5, 12)	10 (6, 18)	12 (7, 25)	11 (5, 22)	0.080
CCI	3 (1, 4)	2 (1, 2)	6 (3, 8)	0.03	2 (1, 4)	2 (1, 2)	3 (2, 5)	1 (1, 1)	0.519	2 (1, 4)	3 (2, 4)	3 (2, 4)	3 (2, 5)	0.035
48-h mortality	0 (0)	1 (33.3)	0 (0)	0.459	3 (15.8)	1 (14.3)	3 (16.7)	0 (0)	0.976	6 (7.2)	3 (11.5)	2 (6.1)	0 (0)	0.75
IHM	0 (0)	2 (66.7)	0 (0)	0.155	5 (26.3)	2 (28.6)	7 (38.9)	0 (0)	0.757	25 (30.1)	8 (30.8)	5 (15.2)	2 (33.3)	0.38

Continuous data were described using the median and interquartile range (IQR), while categorical data were presented as counts and percentages (%). CLs, capillary lactates; DSI, diastolic shock index; FI, fungal infection; GN, Gram negative; GP, Gram positive; IHM, in-hospital mortality; IQR, interquartile range; NC, negative culture; NLR, neutrophil-to-lymphocyte ratio. The *p*-values indicate the statistical significance of differences observed among the groups.

## Discussion

This analysis revealed that CLs and SLs were statistically different according to sepsis etiology and resulted higher in patients who underwent GP infections. Such difference may be attributed to variations in the pathophysiological mechanisms underlying these infections (11). Although GN infections often induce a more robust inflammatory response compared to GP, the latter may trigger a host immune response characterized by the release of specific toxins and enzymes, which can affect tissue morpho-functional integrity, impair cellular function, thereby leading to localized tissue injury and necrosis (10). Furthermore, the site of tissue injury is more localized in GP infections compared to the systemic effects seen in GN-related sepsis, thus leading to hyperlactatemia (9). Additionally, some GP bacteria (e.g., *Staphylococcus aureus*) produce toxins known as superantigens, which can hyperactivate immune response and inflammatory cascade, thus enhancing tissue damage and lactate production (11).

The NLR is used as a marker of systemic inflammation and immune response and its variations can reflect differences in the host immune response to different pathogens (8). In GN infections, the host immune response typically involves a robust activation of the innate immune system, particularly neutrophils. Conversely, lymphocytes are key components of the adaptive immune system, responsible for orchestrating specific immune responses to pathogens. In the early stages of infection, lymphocyte counts may temporarily decrease due to migration of lymphocytes from the bloodstream to sites of infection or lymphoid tissues. However, in the case of prolonged or severe infection, lymphocyte depletion may occur as a result of immune dysregulation and apoptosis (8, 10). The combination of increased neutrophils and possible decrease in lymphocytes in GN infections may lead to a high NLR ratio (10). In contrast, GP infections may elicit a different immune response characterized by a less pronounced neutrophilic response and a relative preservation of lymphocyte counts. Therefore, the NLR in GP infections may not be as elevated as that in GN infections (10).

The DSI, calculated as the ratio of heart rate to diastolic blood pressure, is used to assess the hemodynamic instability in critically ill patients (8, 11). Differences in DSI levels may reflect variations in the underlying pathophysiological mechanisms and clinical presentation. In this study, the DSI resulted higher in FI. Usually, FI manifest with a gradual onset and a chronic course, allowing for compensatory mechanisms to maintain an initial (“early phase”) hemodynamic stability. However, as the infection progresses, fungal pathogens can invade blood vessels, leading to endothelial damage, vasculitis, and thrombosis. This vascular involvement, reflected by changes in the DSI, can cause an impaired tissue perfusion and a compromised hemodynamic balance (10). On the other hand, compared to FI, bacterial infections typically present with more acute and often fulminant clinical courses, characterized by rapid onset of symptoms and hemodynamic instability. Bacterial pathogens can produce toxins and trigger systemic inflammatory responses, leading to vasodilation, capillary leakage and distributive shock. In bacterial infections, changes in the DSI may be driven by heart rate and vascular tone abnormalities, rather than being solely attributable to changes in diastolic blood pressure (10). Therefore, in the context of FI, where vascular involvement and tissue ischemia play a prominent role in disease pathogenesis, the DSI may be higher due to elevated heart rate and decreased blood pressure, a combination of effects that also explains the significantly reduced MAP level observed in our study. Conversely, in bacterial infections characterized by distributive shock and systemic inflammatory response, the DSI may not be necessarily elevated (9, 10).

Age is a well-known factor influencing both immune response and metabolic parameters in sepsis (12, 13). Our age-adjusted analysis revealed significant variations in CLs, SLs, DSI, and CCI across different age groups, highlighting the potential influence of age on these biomarkers. Specifically, younger patients (<65 years) with GN infections exhibited significantly higher CCI, while in the 65–80 years age group, SL levels were significantly elevated in GN infections. In patients older than 80 years, CL levels were notably higher in GP infections, and CCI was significantly lower in those with NC. These findings align with previous studies that have demonstrated age-related differences in sepsis outcomes and biomarker levels. For instance, a study reported that older patients tend to exhibit altered lactate kinetics and a dysregulated inflammatory response, which can affect biomarker interpretation (14). Similarly, Ko et al. (15) highlighted the impact of age on sepsis mortality, emphasizing the need for age-specific treatment strategies.

Our results suggested that while age is a significant factor influencing sepsis biomarkers, the observed differences across sepsis etiologies are not solely attributable to age-related changes. This underscores the importance of considering age as a confounding factor in sepsis research and clinical practice. Further studies with larger cohorts are warranted to validate these findings and explore the interplay between age and sepsis-related biomarkers in greater detail.

### Limitations

We acknowledge limitations, specifically: (1) a small sample size from a single-center which hampers the statistical power; (2) the assessment of each tool (in particular CLs and SLs) was performed only once for each patient, precluding lactate clearance measurement possibly useful for further prognostic information; (3) since increased lactate levels may be due to many different conditions with peripheral hypoperfusion, a thorough clinical definition of the underlying disease is necessary before applying a diagnosis of sepsis; (4) age variability among groups may represent a confounding factor influencing biomarker levels. Prior studies have demonstrated that lactate clearance and immune response differ with age (12, 13). However, our statistical analysis adjusted for age-related differences, minimized its potential bias.

In conclusion, CLs and SLs significantly differed according to sepsis etiology and resulted higher in patients with GP infections. Both NLR and DSI vary according to the underlying microbiological etiology of septic patients. Our analysis supports the involved tools to early predict etiology and improve pharmacological management of sepsis. Indeed, they might be clinically useful to tailor antimicrobial treatment options (e.g., empiric addition of anti-fungal treatment) in relation to the various pathogenetic mechanisms. Although our findings highlighted statistically significant differences in lactate levels, NLR, and DSI among different sepsis etiologies, their direct impact on clinical decision-making remains uncertain. Different papers indicated the importance of biomarkers in sepsis (16–19), even though they do not correlate them with specific septic etiologies. Future prospective studies should assess whether these variations influence treatment adjustments, resuscitation strategies, and overall patient outcomes.

## Data Availability

The raw data supporting the conclusions of this article will be made available by the authors, without undue reservation.

## ‘Glossary

ABG: Arterial blood gas analysis
CCI: Charlson Comorbidity Index
CLs: Capillary lactates
DBP: Diastolic blood pressure
DSI: Diastolic shock index
ED: Emergency department
FI: Fungal infection
FiO_2_: Fraction of inspired oxygen
GCS: Glasgow Coma Scale
GN: Gram negative
GP: Gram positive
HR: Heart rate
IHM: In-hospital mortality
IQR: Interquartile range
MAP: Mean arterial pressure
NC: Negative culture
NEWS-2: National Early Warning Score-2
NLR: Neutrophil-to-lymphocyte ratio
P/F: PaO_2_/FiO_2_ ratio
RR: Respiratory rate
SBP: Systolic blood pressure
SLs: Serum lactates
SOFA: Sequential Organ Failure Assessment
SpO_2_: Saturation of peripheral oxygen
WHO: World Health Organization
